# Distribution of extrahepatic congenital portosystemic shunt morphology in predisposed dog breeds

**DOI:** 10.1186/1746-6148-8-112

**Published:** 2012-07-11

**Authors:** Lindsay Van den Bossche, Frank G van Steenbeek, Robert P Favier, Anne Kummeling, Peter AJ Leegwater, Jan Rothuizen

**Affiliations:** 1 Department of Clinical Sciences of Companion Animals, Utrecht University, Faculty of Veterinary Medicine, Utrecht, the Netherlands

## Abstract

**Background:**

An inherited basis for congenital extrahepatic portosystemic shunts (EHPSS) has been demonstrated in several small dog breeds. If in general both portocaval and porto-azygous shunts occur in breeds predisposed to portosystemic shunts then this could indicate a common genetic background. This study was performed to determine the distribution of extrahepatic portocaval and porto-azygous shunts in purebred dog populations.

**Results:**

Data of 135 client owned dogs diagnosed with EHPSS at the Faculty of Veterinary Medicine of Utrecht University from 2001 – 2010 were retrospectively analyzed. The correlation between shunt localization, sex, age, dog size and breed were studied. The study group consisted of 54 males and 81 females from 24 breeds. Twenty-five percent of dogs had porto-azygous shunts and 75% had portocaval shunts. Of the dogs with porto-azygous shunts only 27% was male (*P* = 0.006). No significant sex difference was detected in dogs with a portocaval shunt. Both phenotypes were present in almost all breeds represented with more than six cases. Small dogs are mostly diagnosed with portocaval shunts (79%) whereas both types are detected. The age at diagnosis in dogs with porto-azygous shunts was significantly higher than that of dogs with portocaval shunts (*P* < 0.001).

**Conclusion:**

The remarkable similarity of phenotypic variation in many dog breeds may indicate common underlying genes responsible for EHPSS across breeds. The subtype of EHPSS could be determined by a minor genetic component or modulating factors during embryonic development.

## Background

Congenital portosystemic shunts (CPSS) cause portal blood derived from the gastrointestinal tract and other organs in the splanchnic drainage area to flow directly into the systemic circulation. As a consequence portal blood bypasses the liver and is not subjected to hepatic metabolism [1].

Liver shunts are classified into intra- and extrahepatic shunts, based on the anatomical location. An intrahepatic shunt represents a normal embryologic shunt (ductus venosus) bypassing the umbilical blood along the liver into the heart of the fetus, which did not close after birth [2,3]. In contrast, an extrahepatic portosystemic shunt (EHPSS) is not considered as a normal embryonic connection. The EHPSS represent abnormal functional communications between the embryonic vitelline veins, which form the entire extrahepatic portal system, and the cardinal venous system, which normally contributes to all non-portal abdominal veins [4]. The extrahepatic portal vein develops from the different parts of the vitelline vein, and the vena cava and vena (hemi)azygos develop from the embryonic cardinal vein. Connections between the cardinal and vitelline systems do not occur during any phase of embryonic development [4]. Therefore extrahepatic shunts must be considered erroneous developmental anomalies. Affected breeds may have either intra- or extrahepatic liver shunts; these two types occur very rarely in the same breed [1,5-9] and both sexes were reported to be equally affected [8,10]. Pedigree analyses of intrahepatic shunts of Irish wolfhounds [3,11] and of extrahepatic shunts of Yorkshire terriers [12] and Cairn terriers [10,13] have shown an inherited basis of shunts in these breeds. Besides the Cairn and Yorkshire terriers, a breed predisposition for EHPSS has been reported for Jack Russell terriers [8], Dachshunds [14], Miniature schnauzers [6] and Maltese [15], which also indicates a hereditary background of the disorder in these breeds [10,13]. Test matings in Cairn terriers showed that EHPSS in this breed has a complex, probably polygenic mode of inheritance [10].

Extrahepatic shunts can have a portocaval or a porto-azygous localization. In general, dogs with porto-azygous shunts show milder clinical signs [16]. It is not known whether these different shunt types (portocaval and port-azygos) have a different genetic background. Genes that are responsible for embryonic extrahepatic connections could be defect in both main types of EHPSS (portocaval and porto-azygous). Hence, the occurrence of both shunt types within a breed could indicate a common major (genetic) defect.

This study was performed to evaluate the distribution of extrahepatic portocaval and porto-azygous shunts in different dog breeds with the aim to discover if a common genetic basis for both extrahepatic types is plausible. For this purpose, data of 135 dogs with a single EHPSS were retrospectively analyzed. This survey yielded information with respect to extrahepatic shunt type, breed, average age at diagnosis and dog size. Based on the higher number of portocaval shunts (89%) compared to porto-azygous shunts reported in previous studies [8], an increased amount of portocaval shunts is to be expected in our study. The milder clinical signs in dogs with a porto-azygous shunt [16] could cause a later onset of clinical signs. Therefore we could expect a later age at diagnosis of dogs with this type of shunt within our study population.

## Results

**Figure 1:**
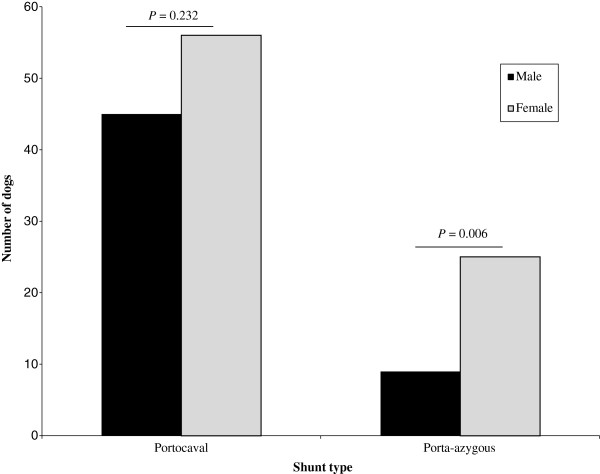
**Localization and gender distribution of extrahepatic portosystemic shunts in 135 purebred dogs.** The dogs were seen in the period from 2001–2010. The shunt diagnosis was based on a high fasting venous ammonia level or abnormal ammonia tolerance test and the visualisation by ultrasonography or computed tomography. In most cases the diagnosis was independently visually confirmed during surgery.

Data of 151 dogs diagnosed with a single EHPSS were available for diagnosis. The 135 dogs used in this study, after excluding 16 cross breeds, were diagnosed with a portosystemic shunt between 6 weeks and 9.7 years of age. Localisation of 93 cases was confirmed during surgery. The study group consisted of 40% males (n = 54) and 60% females (n = 81) which based on t-test was significantly different from the 51% males and 49% females in the total clinic population of 43,813 patients (*P = 0.02*). The proportion of porto-azygous and portocaval shunts in the study group was 25.2% and 74.8%, respectively. By comparing the proportion of males and females between porto-azygous and portocaval we found that in the group of porto-azygous shunts a significantly higher number of females was affected (73.5%) compared with the number of males in the same group (*P* = 0.006). No sex predisposition was found for portocaval shunts (*P* = 0.232) (Figure 1).

**Figure 2:**
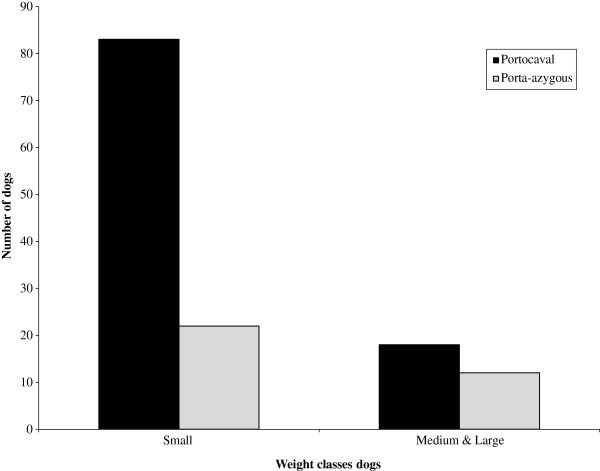
**Weight classification of dogs related to porto-azygous and portocaval shunts.** 135 dogs were classified as small (≤ 9 kilo) or medium and large dog breeds (> 9 kilo). The weight classes contained respectively 101, and 34 dogs. Mean weight is based on the standard of the Dutch Kennel Club. Chi-squared test was performed. A significant difference was observed between weight and shunt localization (*P* = 0.03).

The study group consisted of 24 breeds. In most breeds with six or more cases both porto-azygous and portocaval shunts were diagnosed (Table 1). The only exception were the Pugs which were all affected by portocaval shunts (n = 6). Additional breeds diagnosed with EHPSS were the Lhasa Apso, Miniature Poodle, Norfolk terrier with two cases, and single cases of a Basset Hound, Bolognese, Cavalier King Charles Spaniel, Epagneul Nain Papillon, Flat Coated Retriever, Fox terrier, Giant Spitz, Great Dane, Miniature Pinscher, Norwich terrier and Welsh terrier. In the Cairn terriers a significantly lower fraction of porto-azygous shunts was diagnosed compared to the total study population (*P* = 0.039). The fraction of detected porto-azygous shunts in the other breeds varied considerably, ranging from 0-38% within a particular breed (Table 1). The EHPSS were mainly observed in small dog breeds. Exceptions were a Giant Spitz, a Flatcoated retriever, a Basset hound and a Great Dane. After classification of the dogs as small (≤ 9 kg) or medium and large dog breeds (> 9 kg) these groups contained respectively 101, and 34 dogs. In small dogs portocaval shunts were detected more often than porto-azygous shunts, whereas in the group of medium and large dogs no differences were detected between both types. (*P* = 0.03) (Figure 2).

**Table 1:** Distribution of dogs with congenital extrahepatic portosystemic shunts

**Breed**	**Total**	**M**	**F**	**PC**	**PA**	**Age first diagnosis (months)**	
						**PC**	**PA**
Cairn terrier	24	13	11	22	2	1.5 - 33.2	1.7 - 11.2
Jack Russel terrier	19	8	11	15	4	3.2 - 65.6	4.0 - 113.2
Maltese	15	4	11	13	2	3.0 - 65.6	46.7 - 66.0
Yorkshire terrier	14	8	6	10	4	3.9 - 26.9	3.7 - 32.5
Dachshund	11	6	5	7	4	2.2 - 35.1	6.2 - 65.2
Shih Tzu	9	2	7	8	1	3.7 - 67.4	46.87
West Highland White terrier	8	3	5	5	3	5.0 - 14.0	21.7 - 116.1
Chihuahua	6	2	4	5	1	3.4 - 84.0	10.83
Miniature Schnauzer	6	1	5	4	2	2.9 - 18.9	12.9 - 61.4
Pug	6	2	4	6	0	3.6 - 26.4	NA

M = Male; F = Female; PC = Portocaval shunt; PA = Porto-azygous shunt.

This table only contains purebred dog breeds of which six or more medical records could be included.

A significant difference in the age at first diagnosis was found between the two shunt subtypes (*P* < 0.001). Portocaval shunts were first diagnosed at a mean age of 12.3 ± 11.3 months, whereas porto-azygous shunts were first diagnosed at a mean age of 32.3 ± 24.0 months. All Cairn terriers (n = 24) were excluded from this calculation because most of them were identified by a screening program of pups at an age of six weeks. No significant difference was found in the age at first diagnosis between male and female dogs in porto-azygous shunts (*P* = 0.831) and portocaval shunts (*P* = 0.800).

## Discussion

In our study group of dogs with EHPSS there were significantly more females than males. This is in contrast with previous studies in which an equal sex representation was found [8,10]. Only one breed, the Bichon Frise, has been reported to have an overrepresentation of females with EHPSS [8]. The total population of dogs presented at Utrecht University Clinic between 2001 and 2010 consisted of 49% females and 51% males, indicating that the sex differences found in this study are not caused by differences in the clinic population. In our population the breeds that contributed most to the female over-representation were the Maltese and Shih Tzu. The significantly higher proportion of females with a porto-azygous shunt compared to males with this type of shunt is surprising since we expected a similar sex distribution within the groups of both portocaval and porto-azygous shunts.

In the study group a lower proportion of porto-azygous shunts in comparison with portocaval shunts was found. These findings are well in agreement with previously reported fractions (11-36%) [8,13,15]. Especially in smaller dogs a significant higher frequency of portocaval shunts was detected compared to dogs weighing more than 9 kg. Another observation in line with this is the significantly lower age at first diagnosis of portocaval shunt in comparison with porto-azygous shunts which corresponds with previously published data [17,18]. In porto-azygous shunts, probably less blood bypasses the liver in comparison with portocaval shunts, because the receiving azygos vein has a smaller diameter and therefore more resistance than the abdominal vena cava. Another explanation reported for the later onset of clinical signs in these dogs could be that respiration causes diaphragmatic compression during which the shunt is intermittently closed [16]. Therefore it can be expected that liver functions are better and hepatic encephalopathy less pronounced in dogs with porto-azygous shunts. It is also possible that the later onset of clinical signs could have led to an underestimation of the prevalence of porto-azygous shunts [17,18] especially since with milder clinical signs owners might prefer to go to a referral centre. In this respect it is noteworthy that in the Dutch population of Cairn terriers, which are screened routinely for shunts at the age of six weeks, the fraction of porto-azygous shunts is also low (Table 1). Possibly this overrepresentation might be due to genetic selection on certain phenotypic characteristics and thus being caused by an inbred genetic component. The fact that dogs with porto-azygous shunts are usually diagnosed at an older age increases the risk that they contribute to reproduction and therefore sustain presence of underlying genes in the population. Therefore screening of breeds at-risk at young age seems essential for the extirpation of CPSS in these populations. Only the Dutch Cairn terrier club mandates the test for presence of a shunt in newborn dogs. This could cause a skewed picture of the problem in small dogs. We therefore decided to discard them from analysis of age of diagnosis. The remaining 111 dogs used for this study originate from a diverse population originating from both rural and urban areas.

It should be noted that for a number of breeds only a small number of case reports are available making it hard to draw breed related conclusions on distribution. Localisation of most shunts in the abdominal cavity or the thoracic cavity was confirmed during surgery (n = 93). Because the terminus is not visualized in all cases, a small fraction of the shunts could be wrongly classified. Ultrasound classification using standardized protocols [19] on the other hand proved highly sensitive and specific for diagnosing and classifying EHPSS.

Data of higher numbers of cases would allow us to perform pedigree analysis and also increase the power to detect possible differences in occurrence of shunt types within breeds.

Cairn terriers [10], Yorkshire terriers [12], Jack Russell terriers [8], Dachshunds [14] and Maltese [15] have been described in literature as breeds with a predisposition for EHPSS. These breeds were well represented in our study group. Nearly all dog breeds in our study seem to display both portocaval and porto-azygous shunts. The absence of porto-azygous shunts in pugs is most likely caused by the low number of cases. The fact that the two types affect the same dog breeds has not been reported previously. The occurrence of both porto-azygous and portocaval shunts in nearly all breeds that are predisposed for EHPSS seem to demonstrate that the two types are variants of the same inherited disorder. Modulators like environmental factors during a specific time point in embryogenesis could determine whether the embryonic vitelline system gets erroneously connected with the cardinal vein system at the level of the vena cava or the vena (hemi)azygos. The shunts develop from the portal vein or from one of its contributors, such as the left gastric vein, splenic vein, cranial or caudal mesenteric vein or gastroduodenal vein (vitelline system) [4]. Furthermore, the veins in which the shunts terminate (the caudal vena cava and the azygos vein) are formed through several transformations of the cardinal system [4]. It has previously been shown that EHPSS is a complex genetic trait, presumably determined by different cooperating genes [10]. The observations in our study led to the idea that the two subtypes of EHPSS are commonly determined by a small number of major genes, and that a minor gene or non-genetic factor determines the site of insertion. Future research to these gene defects is needed to confirm this hypothesis. The difference in the prevalence in males and females is yet another puzzle which needs further research.

## Conclusions

In dog breeds predisposed for the occurrence of EHPSS the two shunt types, portocaval and porto-azygous, coexist in nearly all breeds. There seems to be a correlation between location and dog size. Portocaval shunts are predominantly diagnosed in small dogs, whereas no difference was observed in large dogs. The age at first diagnosis in dogs with a porto-azygous shunt is significantly higher than in dogs with a portocaval shunt. This difference is probably a consequence of the lower degree of shunting in porto-azygous shunts resulting in milder clinical signs. Dogs with a porto-azygous shunt may reproduce before diagnosis thereby maintaining causative genes in affected populations. Porto-azygous and portocaval shunts presumably have similar causative genes and are maybe differentiated by a minor genetic component or modulating factors.

## Methods

### Data

Medical records from the University Clinic for Companion Animals of the Faculty of Veterinary Medicine, Utrecht University, the Netherlands, were reviewed to identify dogs with a congenital extrahepatic portosystemic shunt. The following information was retrieved from the medical records: breed, sex, date of birth, localization of the extrahepatic shunt, method of diagnosis and date of first and definitive diagnosis. Data from dogs diagnosed with a single EHPSS in the period 2001–2010 were available for analysis. All cases originated from the Netherlands. Cross breeds were excluded from our study population. The dogs included in this study were presented with clinical signs of hepatic encephalopathy or other signs compatible with portosystemic shunting, or were identified by a shunt screening test performed in the Dutch Cairn terrier population of clinically healthy 6 week old pups. In both cases, a high fasting venous ammonia level or abnormal ammonia tolerance test suggested the presence of a portosystemic shunt [10]. The shunts were visualized by ultrasonography [19] or computed tomography and often confirmed during surgery. The two categories used were portocaval and porto-azygous shunts.

The diagnosis portocaval shunt was made when the shunting vessel terminated in the caudal vena cava. The diagnosis porto-azygous shunt was made when the shunt entered the (hemi)azygos vein, or when the single large tortuous shunting vessel traversed the dorsal part of the diaphragm and was located next to the esophagus. A thoracic termination into the (hemi)azygos vein could not be seen in some cases with ultrasonography or during surgery.

### Statistical method

To assess the relation between breed and extrahepatic shunt localization a Fisher’s exact test was used. To estimate if there was a difference in shunt localization between males and females a Chi-squared test was used for both phenotypes. The Chi-squared test was based on the expected equal distribution of sexes within both shunt types. To determine if there was a correlation between dog size and localization of the shunt, the dog breeds were classified into weight classes. Since the weights of the individual dogs are not comparable due to differences in age and decreased body weight as secondary effect of the phenotype, we used the mean expected weight of the breed based on the breed standard of the Dutch Kennel Club. Dogs were classified as small (≤ 9 kg) or medium and large dog breeds (> 9 kg). A Chi-squared test was performed to estimate if there was a correlation between weight class and localization. In addition, differences in the age at the moment of diagnosis between dogs with portocaval and those with porto-azygous shunts were analyzed with a Mann–Whitney test. In general the age at diagnosis was taken as the moment of the first observation of the presence of a shunt by a high venous ammonia level after fasting or an abnormal ammonia tolerance test. In some dogs the exact localization was determined at a later time point. The difference between age of diagnosis for both groups was calculated performing a t-test. For this calculation the Cairn terrier pups that were diagnosed at young age in a population screening program, and not by clinical signs, were excluded. Also t-test was performed on the age at diagnosis and sex in both phenotypes to estimate a possible sex difference. Significance was considered when *P* ≤ 0.05.

## Competing interests

The author(s) declare that they have no competing interests.

## Authors’ contributions

LvdB and FvS performed the statistical analysis and drafted the manuscript. RF and AK collected data and helped to draft the manuscript. PL and JR conceived of the study, and participated in its design and coordination and helped to draft the manuscript. All authors read and approved the final manuscript.

## References

[B1] Vulgamott JC Portosystemic shunts Vet Clin North Am Small Anim Pract 1985 15 1 229 242 3872504 10.1016/s0195-5616(85)50013-3

[B2] van Steenbeek FG van den Bossche L Leegwater PA Rothuizen J Inherited liver shunts in dogs elucidate pathways regulating embryonic development and clinical disorders of the portal vein Mamm Genome 2011 23 1–2 76 84 22052005 10.1007/s00335-011-9364-0 PMC3275728

[B3] van Steenbeek FG Leegwater PA van Sluijs FJ Heuven HC Rothuizen J Evidence of inheritance of intrahepatic portosystemic shunts in Irish Wolfhounds J Vet Intern Med 2009 23 4 950 952 10.1111/j.1939-1676.2009.0319.x 19496918

[B4] Payne JT Martin RA Constantinescu GM The anatomy and embryology of portosystemic shunts in dogs and cats Semin Vet Med Surg (Small Anim) 1990 5 2 76 82 2196648

[B5] Martin RA Congenital portosystemic shunts in the dog and cat Vet Clin North Am Small Anim Pract 1993 23 3 609 623 8503163 10.1016/s0195-5616(93)50309-1

[B6] Tobias KM Rohrbach BW Association of breed with the diagnosis of congenital portosystemic shunts in dogs: 2,400 cases (1980–2002) J Am Vet Med Assoc 2003 223 11 1636 1639 10.2460/javma.2003.223.1636 14664452

[B7] Winkler JT Bohling MW Tillson DM Wright JC Ballagas AJ Portosystemic shunts: diagnosis, prognosis, and treatment of 64 cases (1993–2001) J Am Anim Hosp Assoc 2003 39 2 169 185 12617545 10.5326/0390169

[B8] Hunt GB Effect of breed on anatomy of portosystemic shunts resulting from congenital diseases in dogs and cats: a review of 242 cases Aust Vet J 2004 82 12 746 749 10.1111/j.1751-0813.2004.tb13233.x 15648933

[B9] Krotscheck U Adin CA Hunt GB Kyles AE Erb HN Epidemiologic factors associated with the anatomic location of intrahepatic portosystemic shunts in dogs Vet Surg 2007 36 1 31 36 10.1111/j.1532-950X.2007.00240.x 17214817

[B10] van Straten G Leegwater PA de Vries M van den Brom WE Rothuizen J Inherited congenital extrahepatic portosystemic shunts in Cairn terriers J Vet Intern Med 2005 19 3 321 324 15954545 10.1892/0891-6640(2005)19[321:icepsi]2.0.co;2

[B11] Ubbink GJ van de Broek J Meyer HP Rothuizen J Prediction of inherited portosystemic shunts in Irish Wolfhounds on the basis of pedigree analysis Am J Vet Res 1998 59 12 1553 1556 9858405

[B12] Tobias KM Determination of inheritance of single congenital portosystemic shunts in Yorkshire terriers J Am Anim Hosp Assoc 2003 39 4 385 389 12873029 10.5326/0390385

[B13] Meyer HP Rothuizen J Congenital portosystemic shunts (PSS) in dogs are a genetic disorder Tijdschr Diergeneeskd 1991 116 Suppl 1 80S 81S 2048089

[B14] van den Ingh TS Rothuizen J Meyer HP Circulatory disorders of the liver in dogs and cats Vet Q 1995 17 2 70 76 10.1080/01652176.1995.9694536 7571284

[B15] Tisdall PL Hunt GB Bellenger CR Malik R Congenital portosystemic shunts in Maltese and Australian cattle dogs Aust Vet J 1994 71 6 174 178 10.1111/j.1751-0813.1994.tb03382.x 8080406

[B16] Sura PA Tobias KM Morandi F Daniel GB Echandi RL Comparison of 99mTcO4(−) trans-splenic portal scintigraphy with per-rectal portal scintigraphy for diagnosis of portosystemic shunts in dogs Vet Surg 2007 36 7 654 660 10.1111/j.1532-950X.2007.00317.x 17894591

[B17] Mehl ML Kyles AE Hardie EM Kass PH Adin CA Flynn AK De Cock HE Gregory CR Evaluation of ameroid ring constrictors for treatment for single extrahepatic portosystemic shunts in dogs: 168 cases (1995–2001) J Am Vet Med Assoc 2005 226 12 2020 2030 10.2460/javma.2005.226.2020 15989185

[B18] Baade S Aupperle H Grevel V Schoon HA Histopathological and immunohistochemical investigations of hepatic lesions associated with congenital portosystemic shunt in dogs J Comp Pathol 2006 134 1 80 90 10.1016/j.jcpa.2005.07.003 16423574

[B19] Szatmari V Rothuizen J Voorhout G Standard planes for ultrasonographic examination of the portal system in dogs J Am Vet Med Assoc 2004 224 5 713 716 698–9 10.2460/javma.2004.224.713 15002810

